# Acupuncture Improves Chronic Cerebral Ischemia by Inhibiting the CKLF1/HIF‐1α/VEGF/Notch1 Signaling Pathway

**DOI:** 10.1111/cns.70246

**Published:** 2025-02-28

**Authors:** Jilong Guo, Guangqi Wang, Tian Liu, Jianjun Zhang, Qinqing Li, Yousong Zhu, Huijuan Luo

**Affiliations:** ^1^ Shanxi Key Laboratory of Chinese Medicine Encephalopathy Jinzhong China; ^2^ Basic Medical College of Shanxi University of Chinese Medicine Jinzhong China; ^3^ Jinzhong City Hospital of Traditional Chinese Medicine Jinzhong China; ^4^ National International Joint Research Center for Molecular Traditional Chinese Medicine Jinzhong China

**Keywords:** acupuncture, cerebral blood flow, chronic cerebral ischemia, CKLF1/HIF‐1α/VEGF/Notch1, cognitive function

## Abstract

**Objective:**

Acupuncture significantly improves cognitive dysfunction in rats with chronic cerebral ischemia. However, the underlying signaling pathways remain unclear. This study investigates the role of the CKLF1/HIF‐1α/VEGF/Notch1 signaling pathway in the acupuncture‐mediated improvement of cognitive function in rats with chronic cerebral ischemia.

**Methods:**

Male SD rats were randomly divided into the normal control group, sham‐operated group, 2‐VO model group, 2‐VO + acupuncture group, and 2‐VO + Ginaton group ( *Ginkgo biloba* extract 14.4 mg/kg/day), with 10 rats in each group. The 2‐VO + acupuncture group received acupuncture at the Shuigou, Baihui, bilateral Fengchi, and bilateral Zusanli points for 14 consecutive sessions over 2 weeks. The rats' memory function was assessed using the Morris water maze and novel object recognition tests. Cerebral blood volume changes were measured using laser speckle imaging. Ultrastructural changes in microvessels were observed via transmission electron microscopy. Neuronal and myelin alterations were evaluated using HE staining, Nissl staining, and LFB myelin staining. The expression levels of CKLF1, CCR5, HIF‐1α, VEGF, and Notch1 proteins were measured using Western blot, and multiple immunofluorescence staining was performed to assess the colocalization of CKLF1 and neurons.

**Results:**

Compared with the 2‐VO model group, acupuncture treatment reduced the latency period and increased the number of platform crossings in the Morris water maze test, and the 2‐VO model group had a higher recognition index in the novel object recognition test. We found that acupuncture improved the condition of endothelial cells, repaired the morphology of the vascular lumen, and alleviated astrocyte edema. We also showed that acupuncture could ameliorate pathological damage in rats with chronic cerebral ischemia. Moreover, acupuncture reduced the expression of CKLF1, CCR5, and HIF‐1α proteins in the hippocampus, decreased the fluorescence intensity of CKLF1 expression, and increased the fluorescence intensity of neurons in the hippocampal CA1 region.

**Conclusion:**

Acupuncture may exert neuroprotective effects and improve cognitive dysfunction caused by chronic cerebral ischemia by regulating the CKLF1/HIF‐1α/VEGF/Notch1 pathway to inhibit inflammatory factors and increase cerebral blood flow.

## Graphical abstract

Acupuncture improves cognitive dysfunction caused by chronic cerebral ischemia by regulating the CKLF1/HIF‐1α/VEGF/Notch1 pathway to increase cerebral blood flow.

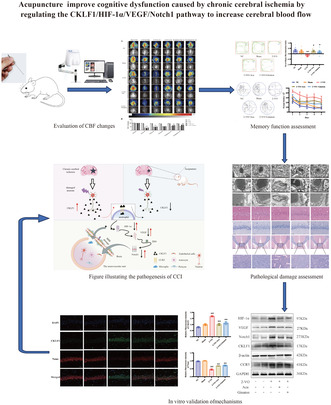

## 1 Introduction

Chronic cerebral ischemia (CCI), also known as chronic cerebral circulation insufficiency or chronic cerebral hypoperfusion, is a cerebrovascular disease initiated by prolonged vascular lesions or circulatory disorders, resulting in reduced cerebral blood supply and subsequent neuronal damage in specific brain regions [1]. CCI damages the cerebrovascular structure, and when cerebral blood flow is insufficient, the brain undergoes decompensation, precipitating a series of chronic, fluctuating clinical syndromes, including lethargy, cognitive dysfunction, or depressive behaviors [2, 3, 4]. As the condition progresses, it may evolve into vascular dementia or Alzheimer's disease [5]. In recent years, epidemiological data have demonstrated that the incidence of CCI is substantial across different age groups: two thirds of cases occur in individuals aged 65 and above, 50% in those aged 50–65, and approximately 25% in individuals aged 45–50. The affected population is gradually shifting toward middle‐aged individuals, rendering CCI an insidious threat to the health of middle‐aged and elderly populations [6]. Due to its high incidence, complex mechanisms, and poor prognosis, CCI imposes a significant psychological stress and economic burden on patients, their families, and society [7, 8, 9]. Consequently, investigating novel therapeutic strategies for CCI and related disorders holds considerable scientific and clinical significance.

The precise regulation of cerebral blood flow (CBF) is crucial for ameliorating CCI. The neurovascular unit (NVU) is the core mechanism of CBF regulation [10]. The physiological function of the NVU is intricately linked with “Qi” and blood [11]. The physiological regulation of CBF primarily depends on the dynamic interaction between the neurons and blood vessels of the brain, with the NVU serving as the structural foundation. Pathological damage to the NVU has emerged as a focal mechanism of cerebral ischemia [12]. Harder posited that the NVU consists of neurons, astrocytes, microglia, endothelial cells, pericytes, and oligodendrocyte precursor cells, all of which are intimately interconnected to form an efficient system for CBF regulation [13, 14]. Based on previous research findings from our team [15, 16, 17], Chemokine‐like factor 1 (CKLF1) may be involved in the physiological activities of the nervous system. CKLF1 is a central transcription factor of inflammatory mediators. It is minimally expressed in the normal adult brain but is significantly upregulated in neurons postischemia. Elevated CKLF1 expression can exacerbate ischemic brain injury. Additionally, CKLF1 exhibits diverse biological activities, colocalizes significantly with hypoxia‐inducible factor 1α (HIF‐1α) in ischemic brain regions, and can further activate HIF‐1α transcriptional activity through C–C motif chemokine receptor 5 (CCR5), thereby creating a positive feedback loop that aggravates blood–brain barrier damage.

During inflammation, the expression of HIF‐1α is often upregulated, leading to endothelial dysfunction and promoting the progression of inflammation [18]. Vascular endothelial growth factor (VEGF) is a neurovascular protein that is expressed in neurons, astrocytes, macrophages, and vascular endothelial cells following cerebral ischemia [19]. The Notch signaling pathway is involved in various processes related to endothelial cell proliferation and survival [20]. These studies suggest that CKLF1, HIF‐1α, VEGF, and Notch1 are potential therapeutic targets for central nervous system injuries, including CCI, and are closely associated with the NVU. However, research conclusions remain inconsistent. Some studies indicate that activating the HIF‐1α/VEGF/Notch1 pathway during cerebral ischemia treatment can promote angiogenesis and restore blood flow [21, 22]. Conversely, under inflammatory conditions, downregulating the HIF‐1α/Notch1 pathway may alleviate inflammation‐induced damage and facilitate neuronal recovery [23, 24]. These findings highlight the need for further research to validate these mechanisms.

*Ginkgo biloba* extract, commonly referred to as Ginaton, is one of the oral medications frequently used in conventional Western medicine and is recognized for its effects on enhancing blood circulation, improving blood flow, and dilating blood vessels [25]. Ginaton is primarily employed to treat both acute and chronic stroke [26], as well as chronic conditions such as memory decline, attention deficit, CCI, dizziness, and dementia. However, prolonged use may induce neurological and cardiovascular adverse effects, as well as gastrointestinal discomfort, significantly impacting the quality of life in patients with CCI [27]. According to clinical symptomatology, CCI corresponds to the traditional Chinese medicine (TCM) categories of “vertigo,” “headache,” “dementia,” “insomnia,” and “depression” [28]. The pathogenesis of CCI in TCM involves multiple aspects, such as Qi and blood, yin and yang, and deficiency and excess [29, 30, 31, 32]. Clinically, the most common syndrome type is Qi deficiency and blood stasis [33, 34], with its primary pathological mechanism being Qi and blood deficiency, leading to malnourishment of the “sea of marrow” [35, 36]. Acupuncture, a component of traditional Chinese medicine (TCM), is extensively used in the treatment of cerebrovascular diseases. TCM has established that acupoints with diagnostic and therapeutic effects are critical locations where meridians connect with internal organs [37, 38]. Research indicates that electroacupuncture has a beneficial effect on cognitive function improvement, with early intervention demonstrating efficacy that meets clinical requirements and aligns with TCM principles of early diagnosis and treatment [39]. Acupuncture is globally recognized as an effective treatment for cerebral ischemia. It has been proven to improve cerebral ischemia by regulating various mechanisms, such as inflammatory responses and apoptosis [40, 41, 42, 43, 44]. Compared with traditional drug treatments, acupuncture is favored by patients and clinicians due to its fewer side effects, ease of operation, and relatively low cost, particularly in the long‐term management of chronic cerebral ischemia, where it offers unique advantages [45]. Clinical trials comparing the efficacy of acupuncture and medication in treating patients with CCI have demonstrated that acupuncture significantly alleviates related clinical symptoms, including dizziness, headache, and forgetfulness, demonstrating superior therapeutic effects [3, 44, 46, 47]. Observational studies on the treatment outcomes of cerebral ischemia patients have also revealed that acupuncture improves vertebrobasilar artery blood flow velocity and hemorheological parameters more effectively than medication. It alleviates cerebral ischemic hypoxia, ameliorates neurological damage, and reduces endothelial inflammation in cerebral arteries [48, 49, 50, 51]. Acupuncture can stimulate the development of collateral circulation, enhance angiogenesis in ischemic areas, and improve local cerebral blood flow [52]. Treatment of ischemic rats with acupuncture at the Shuigou point has been demonstrated to significantly increase neuronal expression in the damaged area [53, 54]. Shuigou has the functions of strengthening the lumbar spine and awakening consciousness, making it one of the primary acupoints in Academician Shixueming's “Awakening the Brain and Opening the Orifices” acupuncture method [55]. Baihui, located at the midpoint of the cranial vertex, regulates Qi and blood and balances yin and yang. Fengchi has the functions of awakening the brain, enhancing intelligence, and opening orifices while unblocking yang meridians. These acupoints are often combined to treat conditions such as dementia, stroke, and forgetfulness [56, 57, 58]. Contemporary studies have found that electroacupuncture at Baihui and Fengchi acupoints can improve cerebrovascular function, regulate cerebral blood flow status, and increase cerebral blood volume in cerebrovascular diseases [59]. The Zusanli acupoint is known for treating cardiovascular and cerebrovascular conditions, as well as general weakness, and acupuncture at this point promotes new microvascular formation and collateral circulation reconstruction in the ischemic cerebral region [60, 61, 62, 63].

Previous animal studies have indicated that acupuncture treatment for CCI typically involves the selection of a single or two acupoints for intervention. However, in clinical practice, the treatment of CCI patients often employs a combination of multiple acupoints to more effectively alleviate disease symptoms. Despite this, the underlying mechanisms of multi‐acupoint therapy remain unclear. Consequently, based on the CKLF1/HIF‐1α/VEGF/Notch1 signaling pathway, this study selected four acupoints, Shuigou, Baihui, Fengchi, and Zusanli, to evaluate the therapeutic effects of acupuncture on CCI and to investigate its underlying mechanisms. This research aims to provide a theoretical foundation for the selection of acupoints in the clinical acupuncture treatment of CCI. Additionally, the study seeks to contribute to research models exploring the mechanisms of acupuncture in treating CCI diseases, such as post‐stroke sequelae and vascular dementia, while also providing foundational support for its clinical application.

## 2 Materials and Methods

### 2.1 Animals

Fifty male Sprague–Dawley rats (body mass 200–210 g), SCXK‐certified (SCXK [Beijing] 2019–0010), and the animal qualification certificate number 110324231105846384, were provided by SPF (Beijing) Biotechnology Co. Ltd. The rats were acclimated for 1 week under conditions, maintaining a temperature of 22°C–25°C, relative humidity at 60% ± 10%, with a 12‐h light/dark cycle, and access to food and water ad libitum. All procedures were conducted in strict accordance with the guidelines of Shanxi University of Chinese Medicine's Animal Ethics Committee (AWE202409420).

### 2.2 Drugs and Reagents

Disposable sterile acupuncture needles (Hwato brand, product batch number: 233110, specification: 0.25 mm × 25 mm, Suzhou Medical Appliance Factory Co. Ltd.), *Ginkgo biloba* extract tablets (batch number: HJ20170088, Dr. Willmar Schwabe GmbH & Co. KG, Germany), Isoflurane (batch number: 2023032001, Shenzhen RWD Life Science Co. Ltd.), CKLF1 (batch number: A16528, Abclonal), CCR5 (batch number: A20261, Abclonal), Notch1 (batch number: A7636, Abclonal), VEGFA (batch number: A0280, Abclonal), HIF‐1α (batch number: GB114936, Servicebio), β‐actin (batch number: AC026, Abclonal), GAPDH (batch number: AC001, Abclonal), NeuN (batch number: GB15138, Servicebio), and CKLF1 (batch number: bs‐2394R, Bioss).

### 2.3 Preparation of the 2‐VO Model

Rats were fasted for 12 h and water‐deprived for 4 h prior to surgery. Anesthesia was induced using isoflurane with a flow rate of 3.0 L/min in an induction chamber. Upon reaching the steady state of anesthesia, the rats were placed in a supine position and secured with a rat holder, with isoflurane flow adjusted to 2.0 L/min to maintain stable anesthesia. Subsequently, the skin on the midline of the neck area was shaved with an electric scalpel, and the area was disinfected with alcohol. A midline incision of 2 cm was made on the neck skin, followed by blunt dissection of the underlying tissues to avoid damaging the vagus nerve and trachea. The bilateral common carotid arteries and vagus nerves were exposed and separated. The bilateral common carotid arteries were ligated to achieve complete blood flow obstruction using 5–0 sutures. The incision was closed in layers using 3–0 sutures. Postoperatively, a prophylactic dose of penicillin was applied to the wound site to prevent infection, and the entire procedure was conducted at a temperature of 35°C–37°C [64, 65].

### 2.4 Grouping and Treatment

Model preparation: Fifty male Sprague–Dawley (SD) rats were randomly divided into three groups: a normal control group (*n* = 10), a sham surgery group (*n* = 10), and a model group (*n* = 30). The model group underwent 2‐vessel occlusion (2‐VO) surgery to induce chronic cerebral ischemia. In the Sham group, the skin along the midline of the neck was dissected, and the tissues were bluntly separated without ligating the bilateral common carotid arteries, with other procedures being identical to the model group. Rats in the NC group were raised under standard conditions. After successful modeling, the rats were randomly assigned to three groups: the 2‐VO model group, the 2‐VO + acupuncture group, and the 2‐VO + Ginaton group, with 10 rats in each group. In the 2‐VO + acupuncture group, rats were restrained using a mouse holder after successful modeling.

Based on the commonly used acupuncture point names and localization in the rat acupuncture point atlas [66], the following points were selected: GV26 (Shuigou), GV20 (Baihui), bilateral GB20 (Fengchi), and bilateral ST36 (Zusanli). Hua Tuo‐brand acupuncture needles were used for treatment. The GV26 point was needled first with a 1 mm oblique upward insertion at the lower nasal septum, followed by a 2 mm oblique insertion at GV20, either forward or backward. The GB20 point was needled obliquely 5 mm toward the nasal tip, and ST36 was needled vertically to a depth of 5 mm. The needles were retained for 20 min. Acupuncture was first performed on the 28th day after successful modeling and was administered once daily thereafter.

The 2‐VO + Ginaton group received an intragastric suspension of *Ginkgo biloba* tablet solution (14.4 mg/kg) once daily. The other groups were administered saline once daily for 14 consecutive days.

### 2.5 Laser Speckle Imaging

CBF Value Monitoring: After anesthetizing the rats with isoflurane, the laser speckle imaging system (Model No.: RFLSI; Serial No.: G1132401‐004, RWD Life Science Co. Ltd.) was used to record the CBF perfusion in the frontal lobe for 20 s. The monitoring period included CBF values before and after modeling. During the modeling process, the laser speckle imaging technique was used to detect changes in regional CBF before and after surgery. The CBF signal acquisition area was located between the anterior and posterior fontanelles in the central region of the ischemic skull. The fur on the top of the head was shaved, and the area was disinfected. A midline incision approximately 4 cm long was made using surgical scissors. The scalp was gently separated on both sides, and the periosteum was removed from the skull. The skull was then ground with a drill until cortical blood vessels were visible. After skull grinding was completed, laser speckle monitoring was performed. Baseline blood flow images and 550 nm transparent observation window data were obtained, capturing grayscale and pseudo‐color images of the laser speckle at each time point [67].

### 2.6 Novel Object Recognition Test

The novel object recognition (NOR) test consists of three phases: the habituation phase, the familiarization phase, and the testing phase. During the habituation phase, each animal is placed in the test chamber for 10 min to acclimate. In the familiarization phase, two identical objects (Objects A1 and A2) are placed in symmetrical positions within the test chamber. The animal is then introduced into the chamber from a position equidistant from the objects, and the recording device is activated. The animal is allowed to freely explore the chamber for 5 min. In the testing phase, 1 h later, Object A1 is replaced with a new object (Object B1) that is similar in size but differs in color and shape. The animal's activity is recorded for another 5 min. The animal's learning and memory capacity are assessed using the NOR index, calculated as follows:

$$\begin{array}{c} \left(\text{Time exploring}\;\mathrm{new}\;\text{object}-\text{Time exploring}\;\mathrm{old}\;\text{object}\right)\\ /\left(\text{Time exploring}\;\mathrm{new}\;\text{object}+\text{Time exploring}\;\mathrm{old}\;\text{object}\right)\\ \times 100\%. \end{array}$$

### 2.7 Morris Water Maze

The Morris Water Maze consists of a black circular tank with a diameter of 1.5 m and a height of 0.5 m, segmented into four quadrants, with the water temperature maintained at 22°C–25°C. A 10 cm diameter platform is fixed to II quadrant, resting 1 cm below the horizontal plane.

① Spatial Navigation Training Experiment: One day prior to the formal experimentation, all rats are placed in the tank to acclimate to the maze environment for 1 min. The water maze experiment spans 6 days. At the commencement of the experiment, the platform is positioned in a fixed quadrant (quadrant II), with rats being placed within the water tank toward the wall from starting points in quadrants III and IV. The time taken by each rat to locate the platform (escape latency) is recorded. If the rat fails to locate the platform within 60 s, it is guided to the platform, with the latency recorded as 60 s.

② Spatial Exploration Test: This test commences on the sixth day of the spatial navigation training experiment. After removing the platform, rats are placed within the water tank from quadrant IV, facing the wall. The initial time interval it takes for the rat to reach the location where the platform was previously positioned and the number of times the rat crosses the original platform location within 60 s are recorded.

### 2.8 Transmission Electron Microscopy

Rats were anesthetized with isoflurane gas, and their brains were extracted. Using a sharp razor blade, the hippocampal CA1 region tissue was rapidly dissected, with a cerebral sample of approximately 1 mm in size. The tissue was fixed in 1% osmium acid prepared in 0.1 M phosphate‐buffered solution (PB, pH 7.4), followed by dehydration, embedding, and ultrathin sectioning. The microvascular structural pathological alterations in the brain tissue were then observed using transmission electron microscopy.

### 2.9 Hematoxylin & Eosin (HE) Staining

Paraffin‐embedded rat brain tissue sections are dewaxed and then sequentially immersed in gradient alcohols for 1 min each. The sections are then rehydrated for 1 min and rinsed with PBS. Hematoxylin staining is performed for 20 min, followed by a 20‐min rinse in tap water for bluing. Eosin staining is carried out for 2 min, after which the sections are rinsed with PBS. The sections are then dehydrated through gradient alcohols and mounted with a neutral resin. The hippocampal tissue structures of the rat brain are observed under a microscope for morphological changes.

### 2.10 Nissl Staining

After dewaxing rat brain tissue paraffin sections, rinse them in distilled water. Dry the sections and outline the tissue with a wax pen. Apply Nissl staining solution and incubate for 10 min. Rinse with distilled water for 2–3 additional times. Dehydrate the sections by sequentially immersing them in ethanol solutions of 75%, 85%, 95%, absolute ethanol, and dewaxing solution for approximately 10 s each. After removal, dry the sections and mount them with neutral resin.

### 2.11 Luxol Fast Blue (LFB) Myelin Staining

Rat brain tissue was collected and embedded in paraffin, followed by sectioning into coronal slices with a thickness of 20 mm. Sections from the same plane were selected for each group. The brain slices were dewaxed and then subjected to myelin staining until the myelin appeared blue against a near‐colorless background. Subsequently, the sections were counterstained with eosin, dehydrated, and finally mounted with neutral resin. Microscopic examination and image acquisition analysis were performed to assess the staining results.

### 2.12 Immunoblot Assay

The total protein was extracted from tissue cells, and the protein concentration of each group was quantified using the BCA assay. Loading buffer was added to the protein samples and mixed thoroughly, followed by boiling for 10 min. A total of 10 μg of protein was loaded per sample, separated via electrophoresis, and transferred onto PVDF membranes. Membranes were blocked at room temperature for 1 h. Primary antibodies were diluted in antibody dilution buffer at a ratio of 1:1000 and incubated overnight at 4°C. Subsequently, the membranes were incubated with appropriately diluted secondary antibodies (1:10000) at room temperature for 1 h. Chemiluminescent detection was performed using ECL, and the target protein bands were analyzed using ImageJ for grayscale analysis. Quantitative comparisons were then conducted based on the results obtained.

### 2.13 Multiplex Immunofluorescence Staining

Paraffin‐embedded rat tissue sections underwent deparaffinization, antigen retrieval, fixation, and blocking. The sections were incubated with primary antibodies overnight at 4°C. After removing the primary antibodies with PBS washing, secondary antibody incubation was performed at 37°C for 1 h. The sections were then stained with DAPI for 10 min, followed by staining with Tissue Fluorescence Quenching Solution B for 5 min. After a final PBS wash, the sections were mounted and observed under a fluorescence microscope (Model No.: MF43‐N, Carl Zeiss Optical Co. Ltd.). This procedure allows for the visualization of specific proteins and their localization within the brain tissue using fluorescence microscopy.

### 2.14 Statistical Evaluation

All data are expressed as mean ± standard error. GraphPad Prism 9.0 (GraphPad Software, USA) was utilized to analyze the data in this study. The normality of the distribution of continuous variables was assessed using the Shapiro–Wilk normality test. For normally distributed data, Student's *t*‐test (two‐tailed) and one‐way ANOVA by Bonferroni multiple comparison test were employed. Additionally, the Mann–Whitney *U*‐test and Kruskal‐Wallis test were employed to compare two or more groups of non‐normally distributed data, respectively. For the MWM test, two‐way ANOVA followed by Bonferroni multiple comparison test was applied for statistical analysis. *p* < 0.05 was considered statistically significant.

## 3 Results

### 3.1 Experimental Flowchart

**FIGURE 1:**
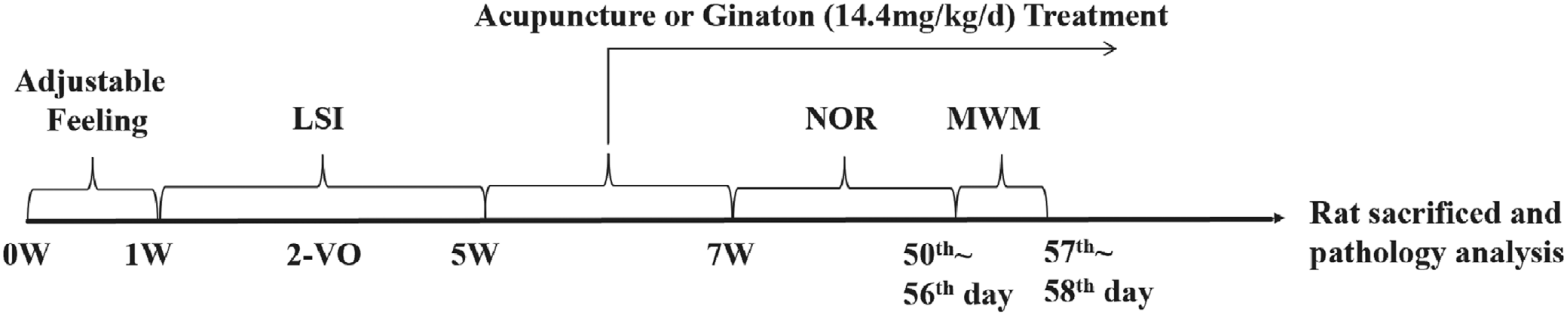
Experimental flowchart.

As shown in Figure 1, the experimental procedures include adaptive feeding, bilateral common carotid artery occlusion modeling, laser speckle imaging to monitor cerebral blood flow, acupuncture, and drug treatments, followed by behavioral testing, transmission electron microscopy, HE staining, Nissl staining, myelin staining, Western blotting, and multiplex immunofluorescence experiments.

### 3.2 Evaluation of CBF Changes in Rats After 2‐VO and Acupuncture Intervention

**FIGURE 2:**
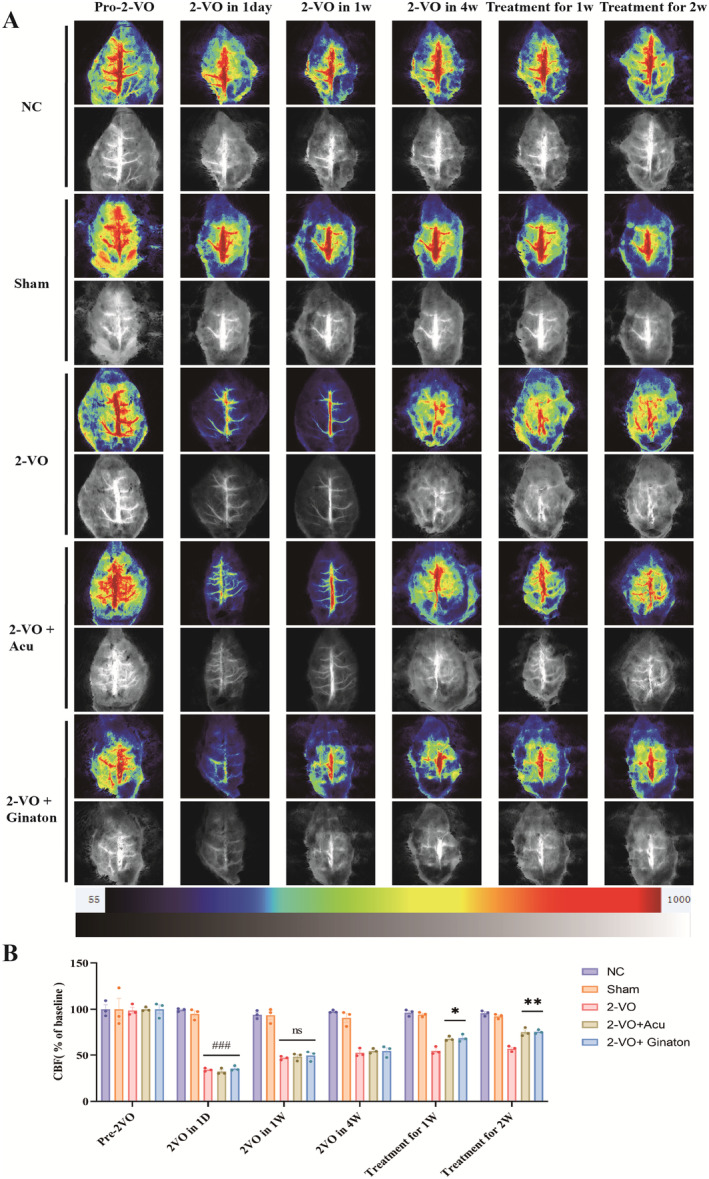
Evaluation of CBF changes in rats after 2‐VO and acupuncture intervention (X¯ ± SEM, *n* = 3). (A) CBF distribution maps for each group of rats obtained using the LSI system; (B) Changes in CBF for each group of rats. Compared with the Sham group, ^###^ *p* < 0.001; comparison with the 2‐VO model group, *p < 0.05, **p < 0.01.

To confirm that the 2‐VO method used to establish the rat model of CCI reduces CBF [68, 69]. As shown in Table 1 and Figure 2A,B, we used the LSI system to measure changes in CBF at different time points (before 2‐VO surgery, 1 day after 2‐VO surgery, 1 week after 2‐VO surgery, 4 weeks after 2‐VO surgery, 1 week posttreatment, and 2 weeks posttreatment) in the rat cerebral artery region. One day after ischemia, the CBF in the Sham group was 95.07% ± 4.83%, whereas the CBF in the 2‐VO model group significantly decreased, with the remaining CBF at 34.20% ± 1.41% (*p* < 0.001). After 1 week of acupuncture treatment, CBF recovered to 68.11% ± 2.06%, compared to 54.93% ± 3.40% in the 2‐VO model group (*p* < 0.05). After 2 weeks of acupuncture treatment, CBF further recovered to 75.15% ± 3.72%, while the 2‐VO model group showed a CBF of 57.24% ± 2.52% (*p* < 0.01). With the increase in treatment duration, CBF within the region of interest (ROI) showed significant recovery (*n* = 3/group).

**TABLE 1:** Changes in CBF in the ROI of rats at different time points after 2‐VO surgery (X ± S, *n* = 3).

Group	Pre‐2VO	2VO in 1D	2VO in 1 week	2VO in 4 weeks	Treatment for 1 week	Treatment for 2 weeks
NC	100.00% ± 6.85%	99.25% ± 1.34%	94.13% ± 3.57%	97.52% ± 1.17%	96.33% ± 2.92%	95.67% ± 2.45%
Sham	100.00% ± 16.7%	95.07% ± 4.83%	93.57% ± 6.49%	90.75% ± 6.66%	94.34% ± 2.00%	92.40% ± 2.08%
2‐VO	98.81% ± 4.92%	34.20% ± 1.41%	46.90% ± 1.63%	52.66% ± 3.86%	54.93% ± 3.40%	57.24% ± 2.52%
2‐VO + Acupunture	100.00% ± 1.81%	32.17% ± 2.72%	48.36% ± 3.31%	54.76% ± 2.23%	68.11% ± 2.06%	75.15% ± 3.72%
2‐VO + Ginaton	100.00% ± 6.86%	35.61% ± 2.23%	49.56% ± 4.57%	54.76% ± 5.18%	68.81% ± 3.26%	75.56% ± 1.79%

### 3.3 Acupuncture Improves Spatial Memory Impairments in the 2‐VO Model Rats

**FIGURE 3:**
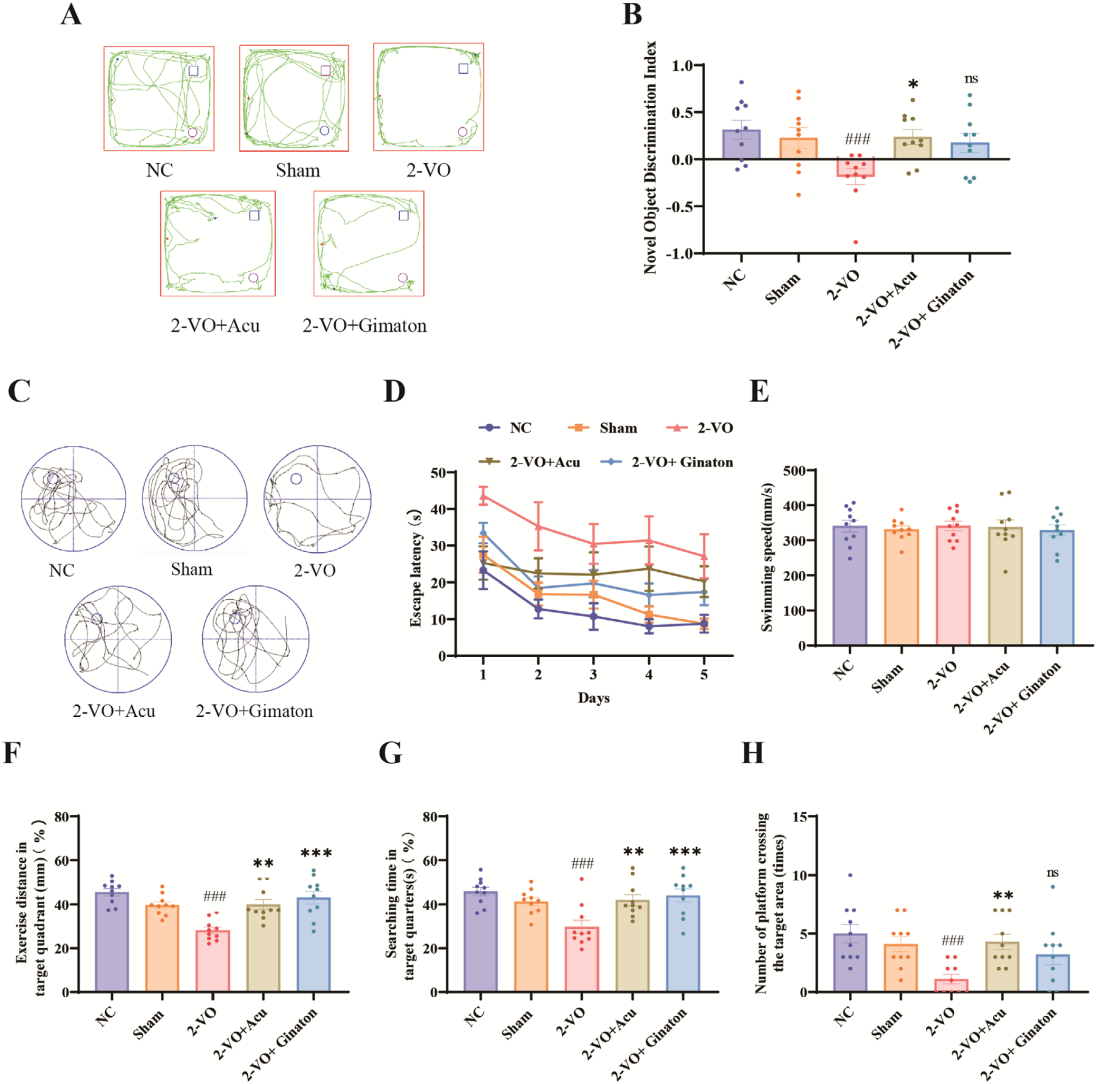
Acupuncture improves spatial memory impairments in the 2‐VO model rats (X¯ ± SEM, *n* = 10). (A) Representative trajectory maps of novel object recognition in each group, (B) statistical results of the novel object recognition index in each group, (C) representative routes of spatial exploration in the Morris water maze test for each group, (D) line chart of the latency in the Morris water maze navigation task for each group, (E) statistical results of swimming speed during the spatial exploration task in the Morris water maze for each group, (F) statistical results of the percentage of distance traveled in the target quadrant during the Morris water maze spatial exploration task for each group. (G) statistical results of the percentage of time spent in the target quadrant during the Morris water maze spatial exploration task for each group, (H) statistical results of platform crossings during the Morris water maze spatial exploration task for each group. Compared with the Sham group, ^###^ *p* < 0.001; compared with the 2‐VO model group, *p < 0.05, **p < 0.01, ***p < 0.001.

During the testing phase of the NOR experiment (Figure 3A,B), the NC and Sham groups of rats exhibited a high level of exploratory behavior and curiosity toward the new object. In contrast, the 2‐VO model rats showed little interest in the replacement of new and old objects, spending most of their time aimlessly wandering in the testing box. After acupuncture and Ginaton treatments, the 2‐VO model rats gradually displayed an increased interest in the novelty of the new object, with the 2‐VO + Acupuncture group showing the most significant curiosity toward the new object. According to the novel object recognition trajectory plots and recognition indices for each group, the recognition index in the 2‐VO model group was significantly lower compared to the sham surgery group (*p* < 0.001). Compared to the 2‐VO model group, the 2‐VO + Acupuncture group showed a significant increase in the novel object recognition index (*p* < 0.05), indicating that acupuncture treatment significantly improved learning and memory capabilities.

The results of the Morris water maze experiment (Figure 3C,D) indicated that, with increasing training days, the time taken by the NC and sham groups of rats to find the submerged platform decreased progressively. In contrast, the 2‐VO model group rats did not show significant changes in the time required to locate the platform with increased training days compared to the sham surgery group. However, the time taken by the 2‐VO + Acupuncture group rats to find the submerged platform decreased to varying degrees with increased training days (*p* < 0.05). During the formal testing phase, statistical analysis revealed no significant differences in swimming speed among the groups (Figure 3E), indicating that there were no notable differences in the motor abilities of the rats (*p* > 0.05). As shown in Figure 3F–H, most rats in the 2‐VO model group swam aimlessly along the edges of the platform, while rats in the normal control and sham surgery groups exhibited purposeful behavior, spending most of their time in the target quadrant and showing platform‐seeking behavior. The 2‐VO + Acupuncture and 2‐VO + Ginaton groups also demonstrated varying degrees of target quadrant platform‐seeking behavior. Compared with the 2‐VO model group, the 2‐VO + Acupuncture group rats showed significant improvement in spatial memory, as indicated by the distance ratio to the target quadrant, search time ratio, and number of platform crossings (*p* < 0.01). These data suggest that the 2‐VO model group rats exhibited memory deficits and abnormal spatial localization abilities, and that acupuncture treatment effectively improved spatial memory deficits in the 2‐VO model rats to some extent.

### 3.4 The Effect of Acupuncture on the Morphological Structure of Microvessels in the

Hippocampal CA1 Region.

**FIGURE 4:**
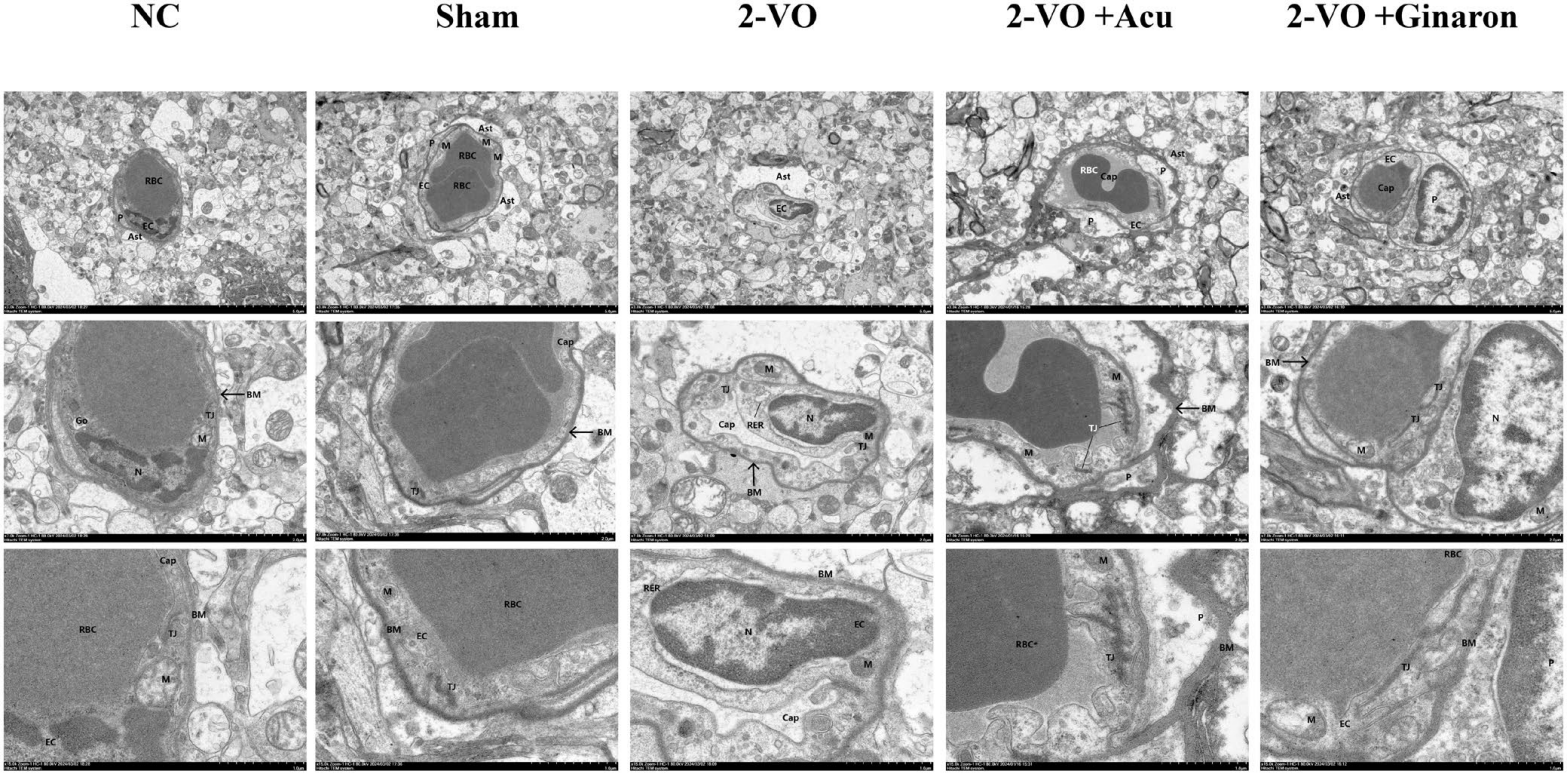
The Effect of acupuncture on the morphological structure of microvessels in the hippocampal CA1 region. Transmission electron microscopy images (×3.0 K, ×7.0 K, ×15.0 K).

Transmission electron microscopy results indicate the following (Figure 4): In the NC and Sham groups, the blood–brain barrier (BBB) exhibits mild damage. Endothelial cells (EC) show slight edema, with a uniform cytoplasmic density and visible tight junctions (TJ). The ECs display a high electron density in a band‐like distribution; the cell nuclei (N) are irregular in shape, with intact nuclear membranes and no widening of the perinuclear space, and chromatin is sparsely aggregated. Mitochondria (M) are slightly swollen with uniform size, intact membranes, slightly pale matrix, shortened cristae, and mildly expanded Golgi apparatus (Go) with intact membrane sacs. Pericytes (P) appear slightly swollen with pale cytoplasm. The capillary lumen (Cap) does not show narrowing or deformation, with a large aggregation of red blood cells. The basement membrane (BM) is intact, continuous, and uniform in thickness. Astrocyte foot processes (Ast) show mild edema and sparse matrix. In comparison with the sham group, the 2‐VO model group demonstrates moderate to severe BBB damage. ECs exhibit edema with uneven cytoplasmic distribution, shorter tight junctions, and relatively wide intercellular gaps. Nuclei are irregular, with intact nuclear membranes and widened perinuclear spaces, with some chromatin aggregation. Mitochondrial membranes are damaged with dissolved matrix, shortened or absent cristae. The rough endoplasmic reticulum (RER) is significantly expanded with dissolved membranes and degranulated surface ribosomes. Capillaries show localized collapse and atrophy. Astrocytes are significantly edematous with extensive matrix dissolution. Compared with the 2‐VO model group, the 2‐VO + Acupuncture and 2‐VO + Ginaton groups show mild to moderate BBB damage following treatment. ECs exhibit slight edema with uniform cytoplasm. Nuclei are less distinct. Mitochondrial structures are relatively intact, with uniform size, intact membranes, and a rich, uniform matrix. TJ structures are abundant with a high electron density band‐like distribution. Pericytes show significant edema with extensive low electron density areas. Capillaries are slightly deformed with large aggregates of red blood cells. The basement membrane is intact and uniform in thickness. Astrocytes show slight edema, membrane damage, and sparse matrix. Thus, acupuncture treatment can improve the state of mitochondria, endothelial cells, and the basement membrane, reduce perivascular edema, repair capillary morphology, and alleviate astrocyte edema.

### 3.5 Acupuncture Alleviates Pathological Damage in CCI Rats

**FIGURE 5:**
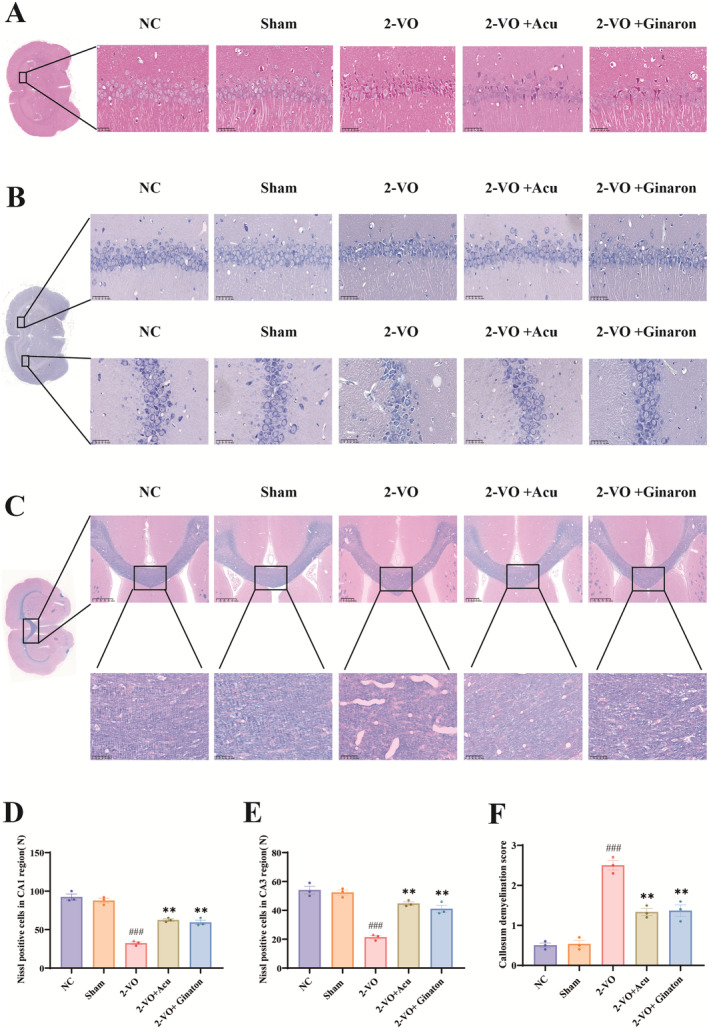
Acupuncture alleviates pathological damage in CCI rats (X¯ ± SEM, *n* = 3). (A) HE staining images of CA1 neuronal cells, (B) Nissl staining images of CA1 and CA3 regions, (C) Myelin loss staining images in each group of rats, (D) quantitative analysis of positive neurons in the CA1 region, (E) quantitative analysis of positive neurons in the CA3 region, (F) scoring results of myelin staining. Compared with the sham group: ^###^ *p* < 0.001, compared with the 2‐VO model group: **p < 0.01.

HE staining results indicate (Figure 5A) that in the NC and Sham groups, CA1 region neuronal cells exhibit intact morphology with a tight and orderly arrangement. The nuclei are large, round, and clearly visible, with even staining and no noticeable swelling or nuclear atrophy. In contrast, the CA1 region neurons in the 2‐VO model group display a disordered arrangement, a reduced number of normal neurons, unclear layering, and abnormalities such as neuronal nuclear condensation, cell body shrinkage and deformation, and deep staining, making it difficult to differentiate normal cell morphology. Compared with the 2‐VO model group, the CA1 region neurons in the 2‐VO + acupuncture group show a significant reduction in abnormal phenomena, an increase in the number of intact neurons, and minimal differences in abnormal neuronal changes. The 2‐VO + Ginaton group also shows a notable reduction in neuronal abnormalities, with a more orderly arrangement, an increased number of neurons, and clearer nucleoli, indicating significant restoration of cell morphology.

Nissl staining results (Figure 5B,D,E) show that in the NC and Sham groups, neurons in the CA1 and CA3 regions are well‐organized and exhibit intact morphology, with abundant Nissl bodies in the cytoplasm. In contrast, the 2‐VO model group rats display a reduced number of neurons in the hippocampal CA1 and CA3 regions, with disordered and scattered arrangement, larger gaps, and a significant decrease in Nissl bodies. This results in vacuolar degeneration, as well as neuronal apoptosis, atrophy, and nuclear dissolution (*p* < 0.001). Compared with the 2‐VO model group, the 2‐VO + acupuncture group shows a reduction in neuronal apoptosis, necrosis, atrophy, and nuclear dissolution with increased neuron count and more Nissl bodies in the cytoplasm as treatment time progresses (*p* < 0.01). The 2‐VO + Ginaton group also demonstrates a tendency toward more orderly neuronal arrangement and relatively intact morphology (*p* < 0.01).

The results of LFB myelin staining (Figure 5C,F) were scored according to the criteria in Table 2 [70], to assess the degree of demyelination in each group of rats. In the NC and Sham groups, myelin morphology appeared normal, with uniform staining and abundant distribution. Compared with the Sham group, the 2‐VO model group exhibited irregular myelin staining, with significant swelling, myelin sheath fragmentation, loss of refractivity, disorganized structure, and notable vacuolization (*p* < 0.001). In comparison with the 2‐VO model group, the 2‐VO + acupuncture group showed reduced myelin swelling, decreased vacuole formation, and increased positive staining expression (*p* < 0.01).

**TABLE 2:** Scoring criteria for degree of demyelinating lesions in brain tissue.

Lesion grading	Degree of lesion
0	Normal nerve fibers and myelin sheath morphology
1	Disordered nerve fibers, irregular staining of myelin sheath
2	Swollen, broken myelin sheath, significant vacuole formation, loss of myelinated nerve fibers, complete myelin degeneration followed by clearance by phagocytes
3	No longer shows positive staining expression

### 3.6 Acupuncture Modulates the CKLF1/HIF‐1α/VEGF/Notch1 Pathway in the Brains of 2‐VO Model Rats

**FIGURE 6:**
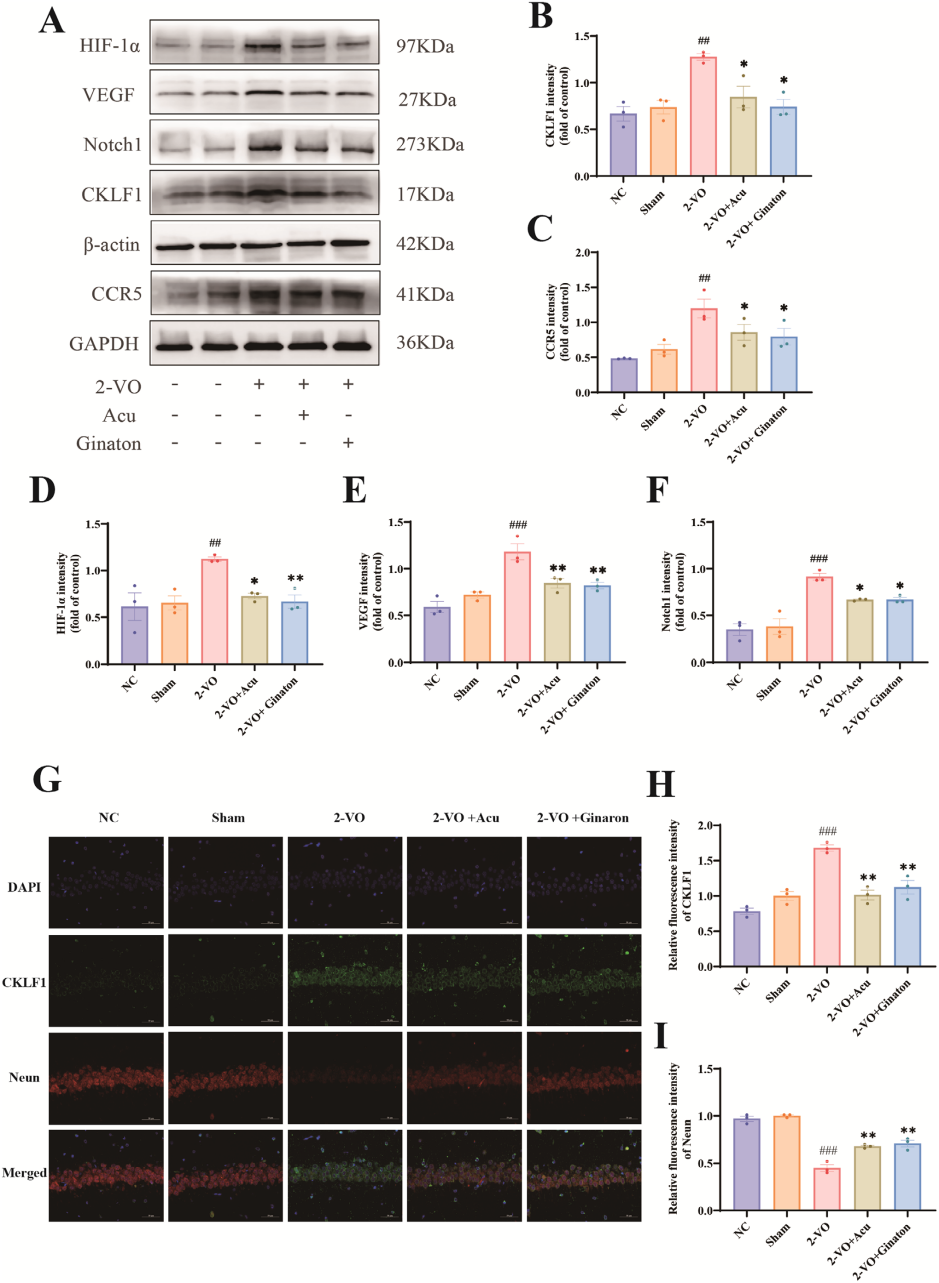
Acupuncture modulates the CKLF1/HIF‐1α/VEGF/Notch1 pathway in the brains of 2‐VO model rats (X¯ ± SEM, *n* = 3). (A) Western blot protein bands, (B) WB statistical analysis of CKLF1 protein, (C) WB statistical analysis of CCR5 protein, (D) WB statistical analysis of HIF‐1α protein, (E) WB statistical analysis of VEGF protein, (F) WB statistical analysis of Notch1 protein, (G) representative micrographs of double‐staining immunofluorescence of CKLF1 (green) with Neun (red) in the CA1 region of the hippocampus. Scale bar: 50 μm, (H) analysis of relative immunofluorescence expression of CKLF1 in the hippocampal CA1 region across groups, (I) analysis of relative immunofluorescence expression of Neun in the hippocampal CA1 region across groups. Compared with the Sham group: ^##^ *p* < 0.01, ^###^ *p* < 0.001; compared with the 2‐VO model group: *p < 0.05, **p < 0.01.

To investigate whether acupuncture alleviates cognitive impairment in 2‐VO model rats via the CKLF1‐related pathway. First, Western blotting was used to detect CKLF1 expression levels in the hippocampus. Acupuncture treatment significantly reduced CKLF1 expression in the hippocampus of 2‐VO model rats (Figure 6A,B) (*p* < 0.05). Next, the expression of CKLF1 receptor protein CCR5 was measured. Acupuncture treatment decreased CCR5 expression in the hippocampus (Figure 6C) (*p* < 0.05). Additionally, the changes in HIF‐1α, VEGF, and Notch1 were further investigated. As shown in Figure 6D–F acupuncture treatment also correspondingly reduced the expression levels of HIF‐1α, VEGF, and Notch proteins in the hippocampus (*p* < 0.05, *p* < 0.01, *p* < 0.05).

In addition, the results of double immunofluorescence staining (Figure 6G–I) demonstrated that, compared with the Sham group, the 2‐VO model group showed a decreased Neun immunofluorescence intensity in the hippocampal CA1 region, whereas CKLF1 fluorescence intensity significantly increased (*p* < 0.001). Compared with the 2‐VO model group, the Neun immunofluorescence intensity in the hippocampal CA1 region of the 2‐VO + Acupuncture and 2‐VO + Ginaton groups was significantly increased, whereas CKLF1 fluorescence intensity was significantly reduced (*p* < 0.01).

## 4 Discussion

In this study, to our knowledge, we are the first to demonstrate that acupuncture may significantly alleviate cognitive impairment and neuronal damage in 2‐VO model rats by modulating CKLF1 and its receptor protein CCR5. The results suggest that CCI not only reduces neuronal survival but also severely disrupts the structure and function of microvasculature. CCI leads to noticeable edema of endothelial cells and astrocytes, as well as matrix dissolution, further contributing to morphological changes in microvessels within the NVU and exacerbating vascular lumen damage, which may underlie cognitive impairment. Acupuncture treatment partially restores these damaged conditions. This indicates that acupuncture not only promotes brain tissue repair by improving blood flow and providing nutritional support but also potentially protects neurons and improves cognitive function by regulating the blood–brain barrier and mitigating inflammatory responses.

CCI is a clinical condition characterized by chronic brain dysfunction caused by a pathological reduction in CBF. While CCI has various etiologies, CCH is a primary factor. CCI is also a common factor in cerebrovascular and cardiovascular diseases and in elderly populations [71, 72, 73]. Prolonged CCI may eventually lead to cognitive impairment [74, 75]. The energy metabolism of brain cells requires continuous delivery of large amounts of oxygen and nutrients through a rich cerebral vascular network [76]. Normal brain function relies on maintaining adequate CBF. However, under pathological conditions such as CCI, it is primarily influenced by factors like abnormal blood pressure fluctuations, atherosclerosis, and increased blood viscosity. Among these, cerebral hypoperfusion caused by low blood pressure is a key factor [6]. As the carotid arteries are major blood vessels supplying the brain, any factors affecting carotid blood flow can result in reduced CBF in patients [77]. Reduced CBF can cause neuronal damage, leading to learning and memory deficits and cognitive decline [4]. In our study, the model group exhibited a substantial decrease in CBF to approximately 34.20% ± 1.41% 1 day after 2‐VO surgery. After 4 weeks, the CBF gradually recovered to about 52.66% ± 3.86%. Following 2 weeks of acupuncture treatment, CBF increased further to 75.15% ± 3.72%. These results suggest that the postoperative reduction in CBF successfully mimics CCI in humans, and acupuncture notably improves the recovery of CBF in rats with CCI. The Morris water maze results showed that acupuncture treatment had a significant impact on the spatial navigation phase. Rats that received acupuncture exhibited a significantly shorter escape latency and a higher number of platform crossings, indicating that the 2‐VO + Acupuncture group was more active in spatial exploration. In the spatial probe test, the 2‐VO + Acupuncture group rats spent more time and covered more distance in the target quadrant, further emphasizing the positive effects of acupuncture on cognitive function and spatial navigation abilities, providing strong support for the improvement of cognitive function. In the novel object recognition test, the Sham group of rats had a recognition index (RI > 0.5), indicating normal memory and a clear interest in exploring new objects. CCI led to a decline in memory abilities in rats, reducing their interest in exploring new objects. After acupuncture treatment, the rats' memory improved, and they spent more time exploring the new objects.

NVU is a core mechanism for the regulation of CBF, and its theory aligns with the traditional Chinese medicine concept of the “holistic view.” This theory particularly emphasizes the critical role of blood vessels in protecting neural structures and functions. According to this theory, blood vessels not only serve as essential pathways for delivering nutrients and oxygen to neurons and other brain cells but also play a vital role in maintaining the health and function of the nervous system. During CCI, the structure and function of the NVU undergo significant changes, such as extensive neuronal apoptosis, damage to the integrity of microvessels, and dysfunction of the blood–brain barrier [78]. These changes directly affect the health of brain tissue, potentially leading to cognitive decline and other neurological issues. Permanent ligation of both common carotid arteries in rats can cause ischemic brain tissue damage, particularly in the hippocampus, which is closely related to learning and memory functions [79]. Our research suggests that acupuncture treatment may alleviate CCI‐induced pathological changes in the brain, including reducing neuronal damage in the hippocampus and mitigating demyelination in the corpus callosum.

Acupuncture is an integral component of TCM, embodying the core concepts of “holism” and “harmony.” It plays a role in regulating internal organs, promoting the flow of Qi and blood, and unblocking meridians while offering advantages such as ease of application [80, 81]. Acupuncture has been shown to possess anti‐apoptotic, anti‐oxidative stress, anti‐inflammatory, autophagy‐regulating, and NVU‐regenerating properties, making it effective in protecting against NVU damage after cerebral ischemia, reducing neurological impairments, and promoting functional recovery [82]. Modern research suggests that acupuncture at the “Shuigou” point not only excites brain neurons, leading to complex integrative effects on the central nervous system, but also improves cerebral blood flow, providing energy for brain cell function recovery—this is the mechanism behind its ability to revive consciousness and regulate mental functions [83]. The “Baihui” point is closely connected to the brain and is key in regulating brain function, with effects that include reviving consciousness, unblocking meridians, enhancing Qi, and nourishing the brain marrow, and improving local blood circulation [84]. Acupuncture at the “Fengchi” point can improve mean blood flow velocity and peak systolic flow velocity in the vertebrobasilar arteries while also regulating vascular endothelial factors and improving vascular tone [85]. The “Zusanli” point, as part of the Yangming meridian that circulates through the eyes and brain, is known for its abundant Qi and blood, and acupuncture here can help to unblock meridians and promote the flow of Qi and blood [86, 87]. Therefore, acupuncture is currently considered an important therapeutic strategy for improving chronic cerebral ischemia [83, 88, 89].

The BBB and CBF are precisely regulated by the NVU to maintain a stable brain microenvironment. Damage to endothelial cells impairs signaling pathways between astrocytes and neurons. Pericytes in the central nervous system contribute to neurogenesis and angiogenesis, and their loss can lead to BBB disruption [90, 91]. Astrocytes regulate CBF by modulating the contraction and relaxation of pericytes and smooth muscle cells (SMCs) [92, 93]. Microglia, as immune cells in the central nervous system, regulate the innate immune response of astrocytes by releasing various signaling molecules [94]. Through transmission electron microscopy, we found that acupuncture treatment helps alleviate BBB damage, improves endothelial cell function, restores vascular lumen morphology, reduces astrocyte edema, maintains basement membrane integrity, and reduces inflammatory responses. This finding is consistent with previous studies [95].

**FIGURE 7:**
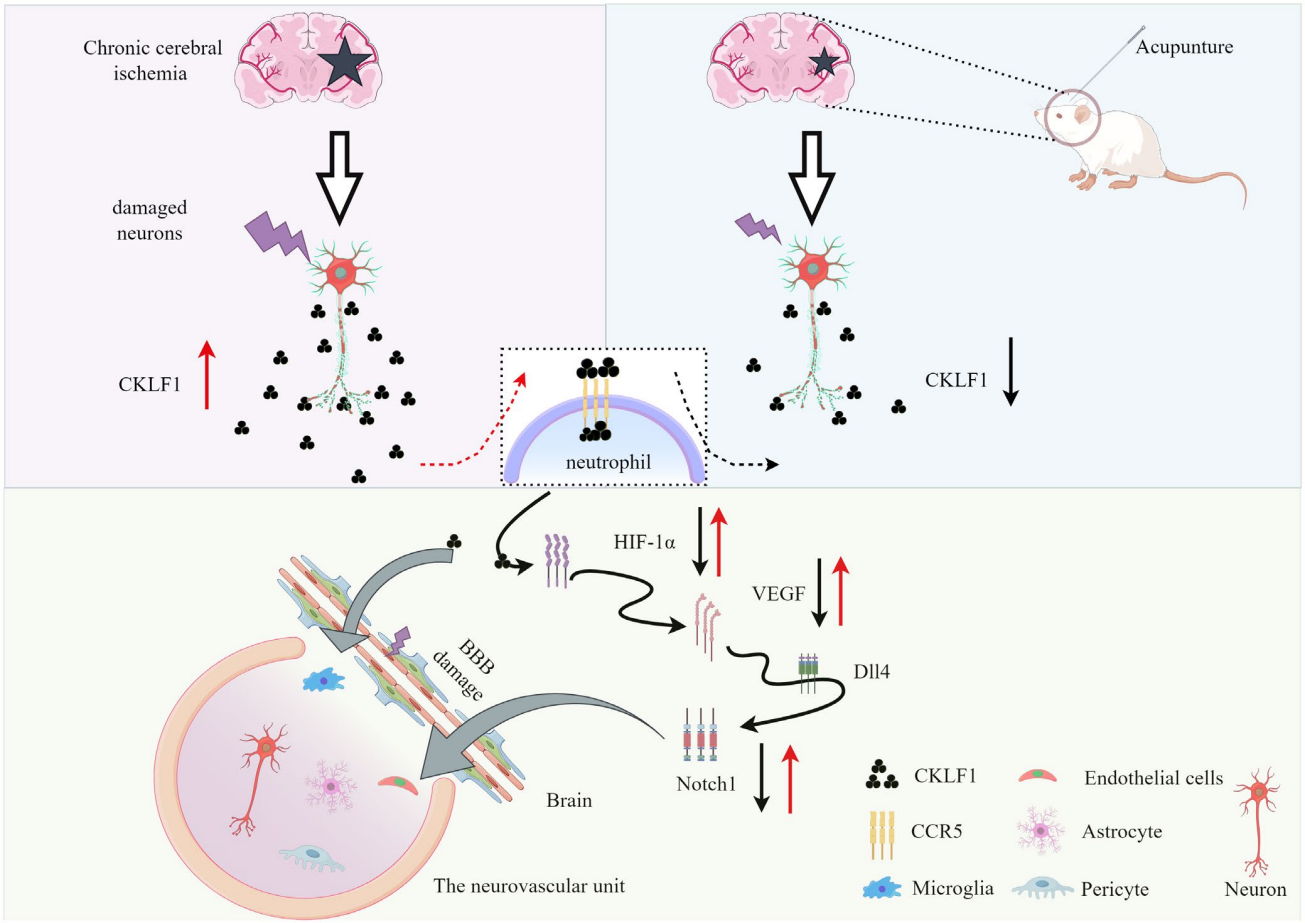
Illustrating the pathogenesis of CCI based on the NVU and the mechanism of acupuncture's therapeutic effects in treating CCI through the NVU (by Figdraw).

The novel chemokine CKLF1 is widely expressed in various tissues and organs in the human body, though its expression is limited in the mature brain or heart [96, 97]. Despite this, CKLF1 may play a significant role in the physiological activities of the brain. Studies have shown that CKLF1, which is produced exclusively in neurons following cerebral ischemia, can exacerbate the inflammatory response to cerebral ischemia [15, 98]. Using multiplex immunofluorescence to detect CKLF1 and neuronal expression, our results align with previous studies, indicating that CKLF1 is expressed in neurons [99]. CCR5 is the primary receptor for CKLF1 [15, 16]. Research indicates that CCR5 expression increases following ischemic stroke [100, 101, 102]. CCR5 is involved in neuroinflammation and plays a critical role in neuroprotection by reducing CCR5 expression [103, 104]. Our experimental results suggest that after the 2‐VO surgery in rats, the expression of the chemokine CKLF1 increased, leading to the release of various pro‐inflammatory factors by neural cells, which in turn triggered an excessive inflammatory response and caused further cellular damage. Acupuncture may reduce CKLF1 and CCR5 expression, thereby decreasing their impact on neuroinflammation and cellular damage, promoting the repair and regeneration of damaged neurons, and protecting neuronal function and the NVU. Additionally, CKLF1 can regulate HIF‐1α through its receptor CCR5 [105]. HIF‐1 is a transcriptional activator composed of a regulatory subunit HIF‐1α and a structural subunit HIF‐1β [106]. HIF‐1α can directly influence the downstream factor VEGF, and the relationship between VEGF and the Notch signaling pathway is associated with changes in endothelial cells [107]. Meanwhile, the Notch signaling pathway is highly conserved in evolution and is closely associated with cell proliferation, differentiation, apoptosis, and the self‐renewal of stem cells. It plays a central role in the regulation of neuronal apoptosis [108, 109]. Studies have found that during cerebral ischemia, γ‐secretase is rapidly activated, leading to an increase in Notch1 levels in the hippocampal region [110]. Activation of Notch1 further promotes the increase in the p65 subunit of the NF‐κB pathway, enhancing the infiltration of inflammatory cells and the expression of inflammatory factors, thereby exacerbating neuronal damage during cerebral ischemia [111]. The Notch pathway increases the vulnerability of neural cells, and inhibiting Notch pathway activation can effectively reduce neural cell damage and brain function loss [112, 113, 114] (Figure 7).

HIF‐1α exhibits dual functions in cerebral ischemia. Studies have found that under normal hypoxic conditions, HIF‐1α is hydroxylated by prolyl hydroxylase and rapidly degraded. However, under hypoxic–ischemic conditions, prolyl hydroxylase activity decreases, preventing HIF‐1α degradation and resulting in its accumulation in the cytoplasm. This accumulation further upregulates the expression of HIF‐1α target genes [65]. Related studies have reported the detrimental effects of HIF‐1α in ischemic brain injury, including severe inflammatory responses and enhanced apoptosis following ischemic stroke, suggesting that HIF‐1α may act as a mediator of neuroinflammation [115]. Consistent with these findings, our results demonstrated increased HIF‐1α expression in the hippocampus of the 2‐VO group compared with the Sham group. In the 2‐VO + Acupuncture group, HIF‐1α protein expression in hippocampal tissue was reduced, indicating a decrease in inflammatory factor expression, promotion of neuronal functional recovery, and repair of damaged brain tissue.

However, HIF‐1α/VEGF/Notch1 is also involved in the expression of various genes associated with neurogenesis, angiogenesis, cell proliferation, erythropoiesis, and cellular metabolism. Studies have demonstrated that activation of the HIF‐1α‐VEGFA‐Notch1 signaling pathway promotes angiogenesis within the cerebral microvascular system, thereby protecting against ischemia–reperfusion injury in the brain [116]. Additionally, HIF‐1α/VEGF/Notch1 participates in processes, such as cell death, adaptive responses, cellular regeneration, and enhancing the brain's resilience to ischemic stress, exhibiting neuroprotective effects [117, 118]. In summary, HIF‐1α/VEGF/Notch1 levels increase following cerebral ischemia but decrease after treatment. Their expression is influenced by the extent of inflammation reduction, neuronal repair, and CBF restoration achieved through acupuncture therapy. This dynamic regulation may partially explain the seemingly paradoxical effects of HIF‐1α/VEGF/Notch1 on the pathological processes of cerebral ischemia.

Our study also has some limitations. First, the exact relationship between the behavioral changes induced by acupuncture and the alterations in signaling pathways remains unclear. Future research could explore whether the behavioral recovery induced by acupuncture can be blocked by reversing the HIF‐1α/VEGF/Notch1 pathway. Second, the effects of different acupuncture point selections, such as Dazhui, Shenting, and Xuehai, and different acupuncture methods, such as embedding needles or electroacupuncture, on this condition have not been fully examined. Future studies could investigate the influence of acupuncture on the CKLF1/HIF‐1α/VEGF/Notch1 signaling pathway by selecting different point combinations or acupuncture techniques.

## 5 Conclusion

In summary, this study targeted the acupoints, Shuigou, Baihui, Fengchi, and Zusanli for acupuncture intervention. Through acupuncture, the expression levels of inflammatory factors such as CKLF1 and CCR5 were effectively inhibited, exerting neuroprotective effects. Meanwhile, acupuncture regulated the HIF‐1α/VEGF signaling pathway, promoted angiogenesis, improved local cerebral blood flow, and optimized the overall regulatory efficiency of the neurovascular unit. This research provides a theoretical basis for acupoint selection in the clinical treatment of CCI and its related diseases. It also explores a potentially safe, economical, and effective intervention for CCI treatment, which is of significant importance in advancing the development of CCI clinical treatment techniques.

## Author Contributions

J.G., Q.L., and J.Z. conceived and devised the experiments; G.W., H.L., and T.L. executed them; G.W., Y.Z., and T.L. tabulated the data and authored the manuscript. All contributors reviewed and approved the final version for publication.

## Conflicts of Interest

The authors declare no conflicts of interest.

## Supporting information


Data S1.

## Data Availability

The data that support the findings of this study are available from the corresponding author upon reasonable request.
