# Supplementary material for: Acupuncture Improves Chronic Cerebral Ischemia by Inhibiting the CKLF1/HIF‐1α/VEGF/Notch1 Signaling Pathway
Source: CNS Neurosci Ther. 2025 Feb 28;31(3):e70246. doi: 10.1111/cns.70246 (PMC11868988; doi:10.1111/cns.70246)

Full unedited gel/blot for Figure 6A

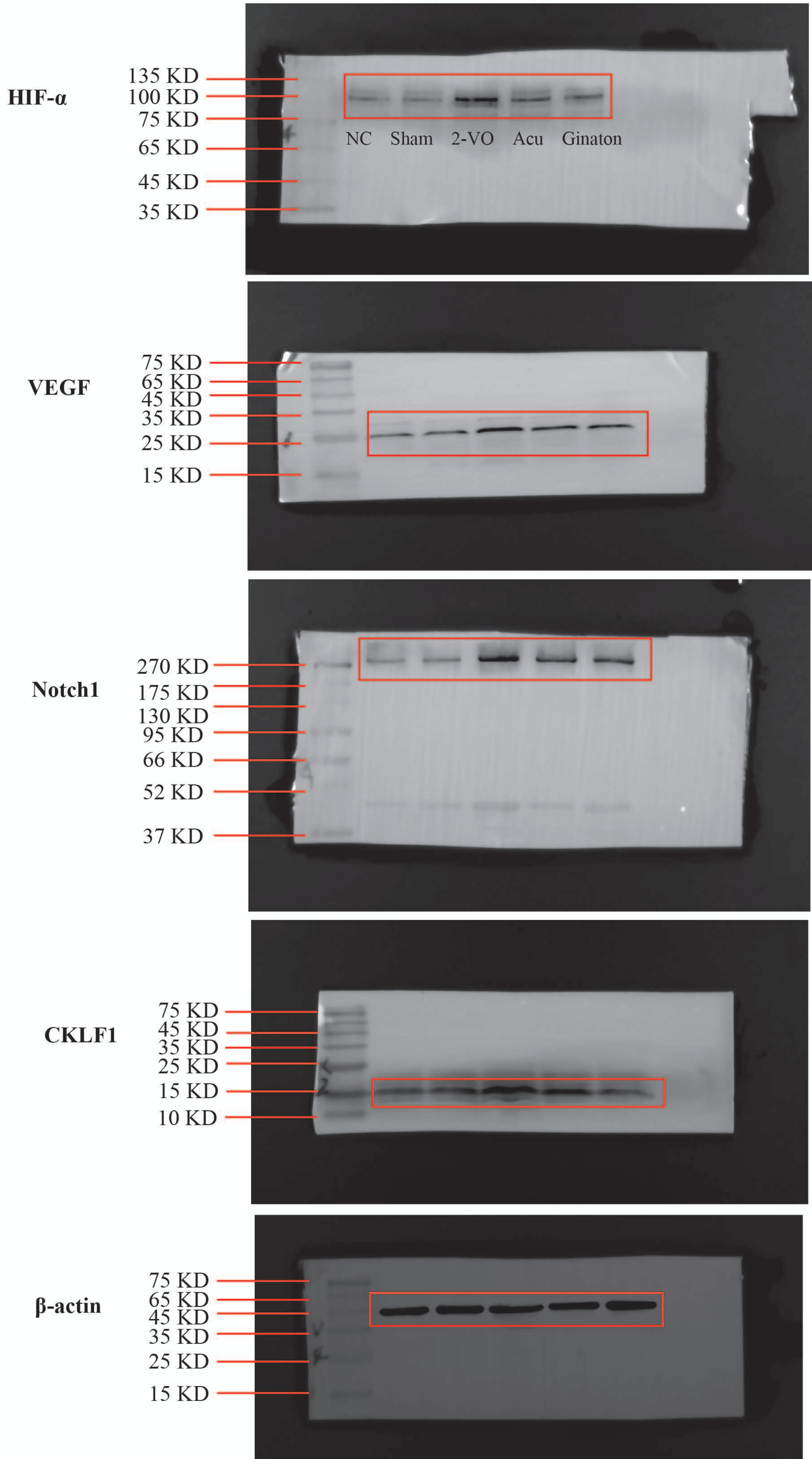

Full unedited gel/blot for Figure 6A

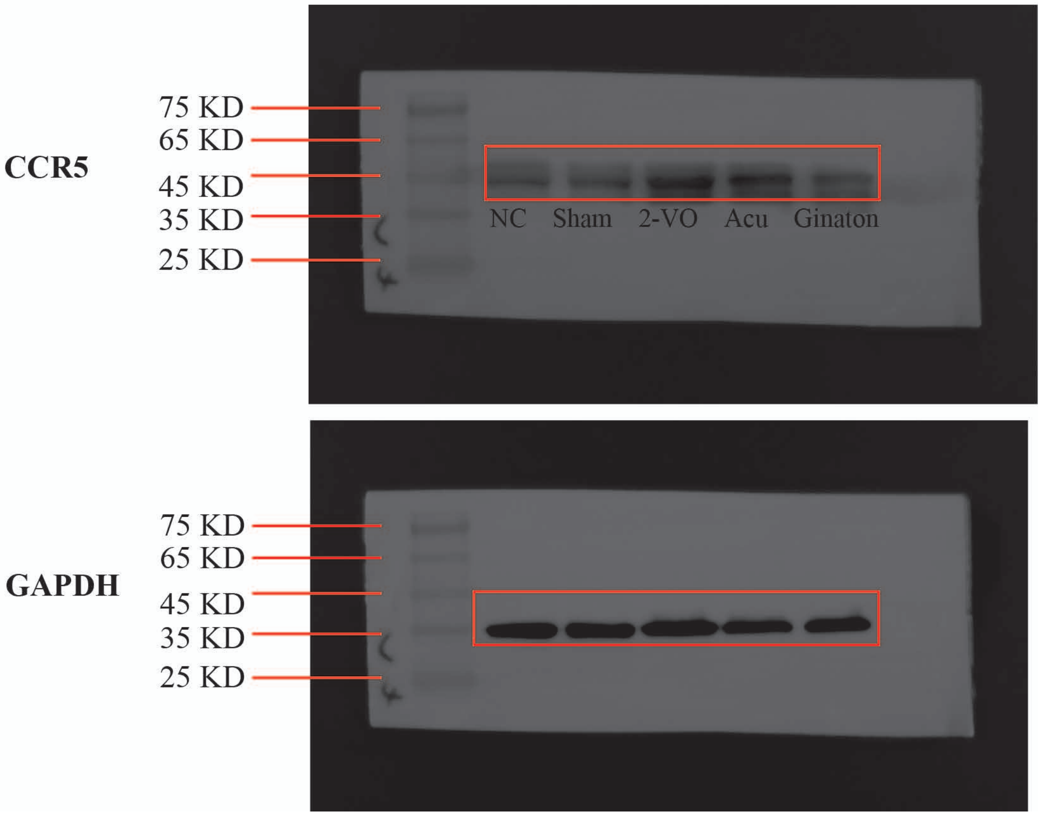

HIF-α

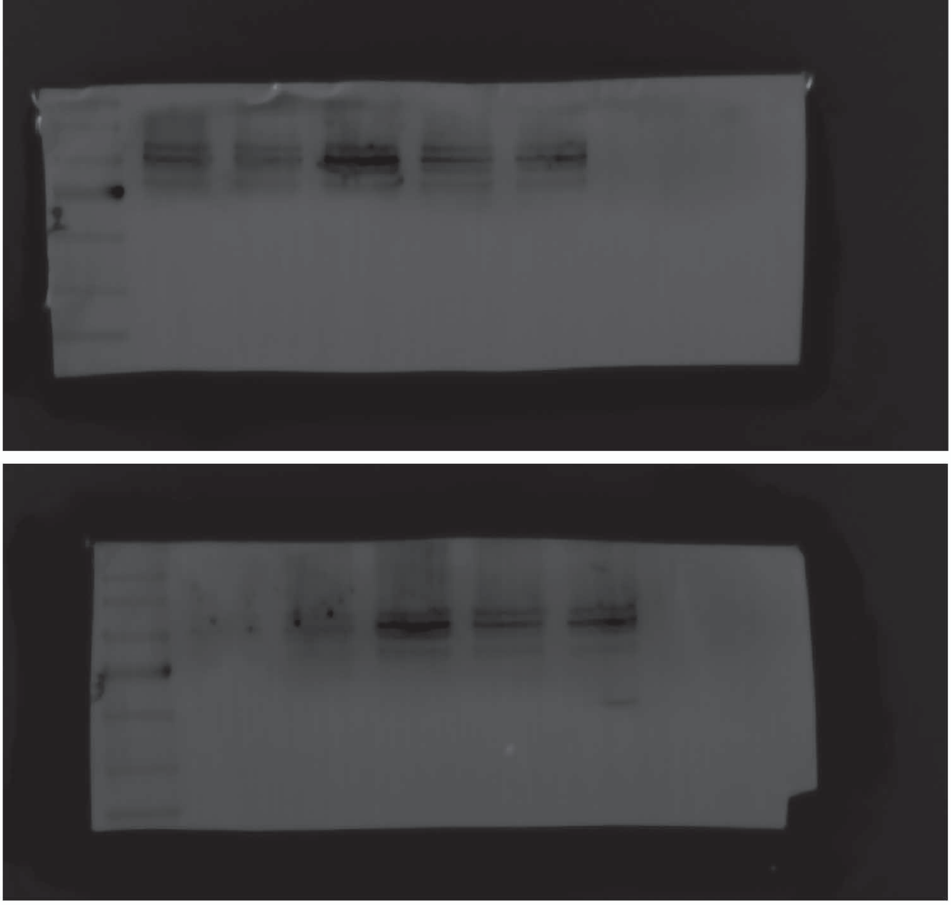

VEGF

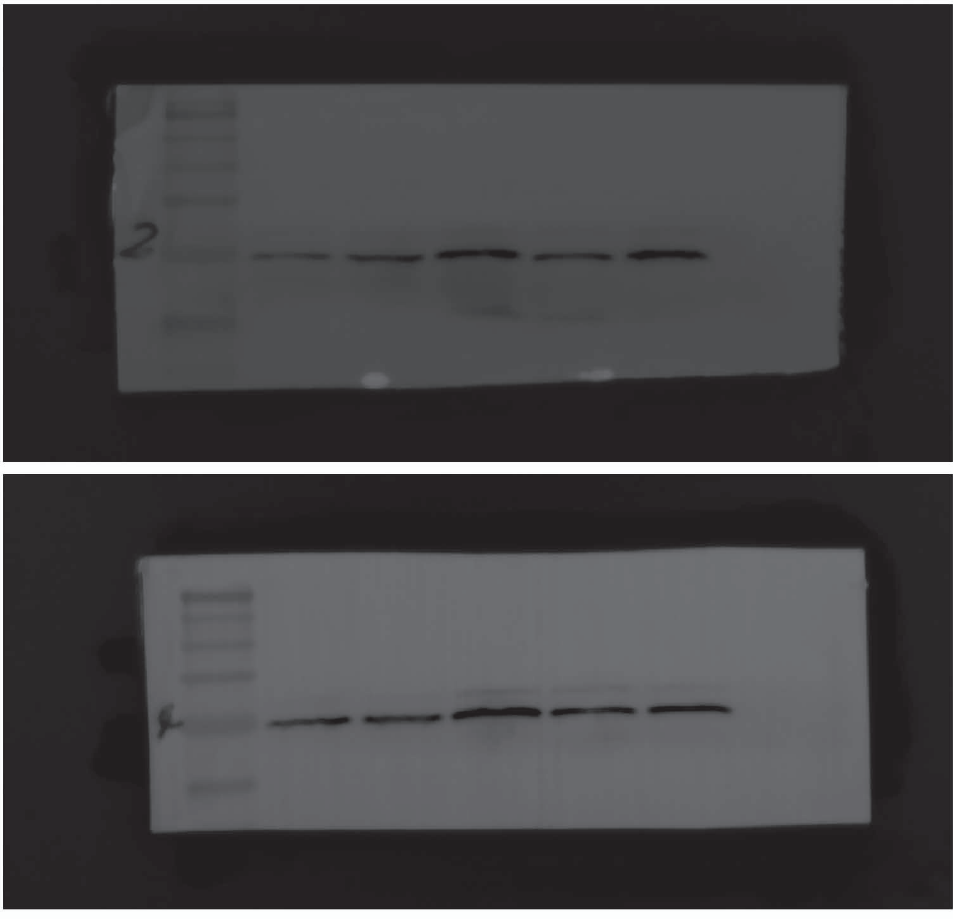

β-actin

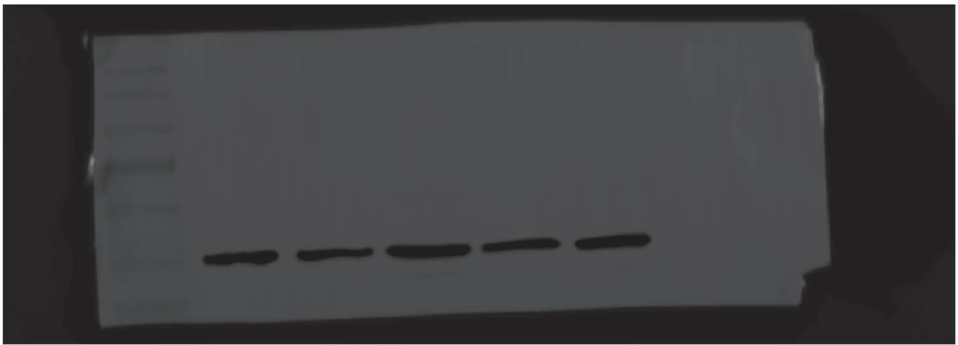

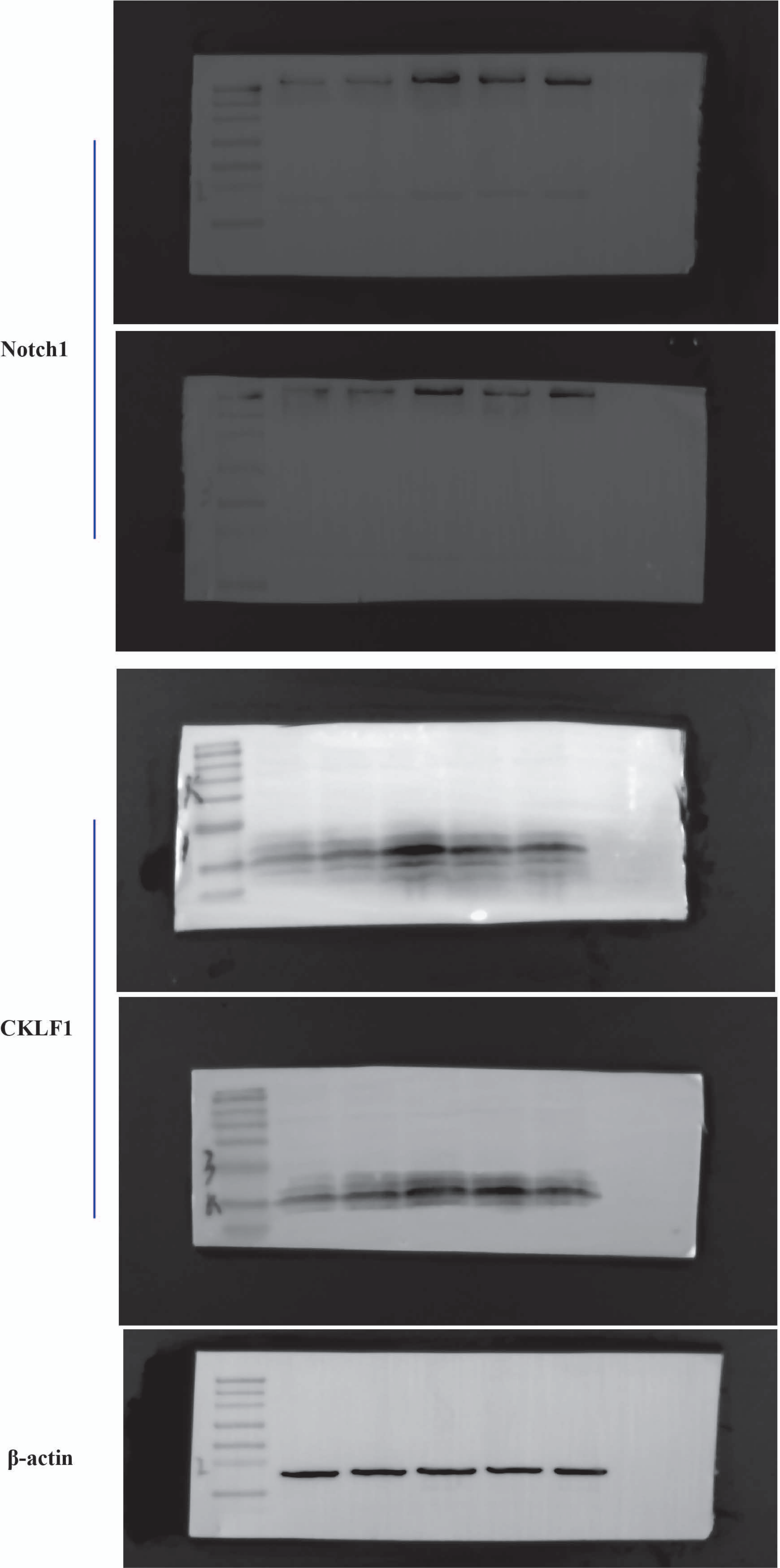
Notch1
CKLF1
β-actin

CCR5

GAPDH

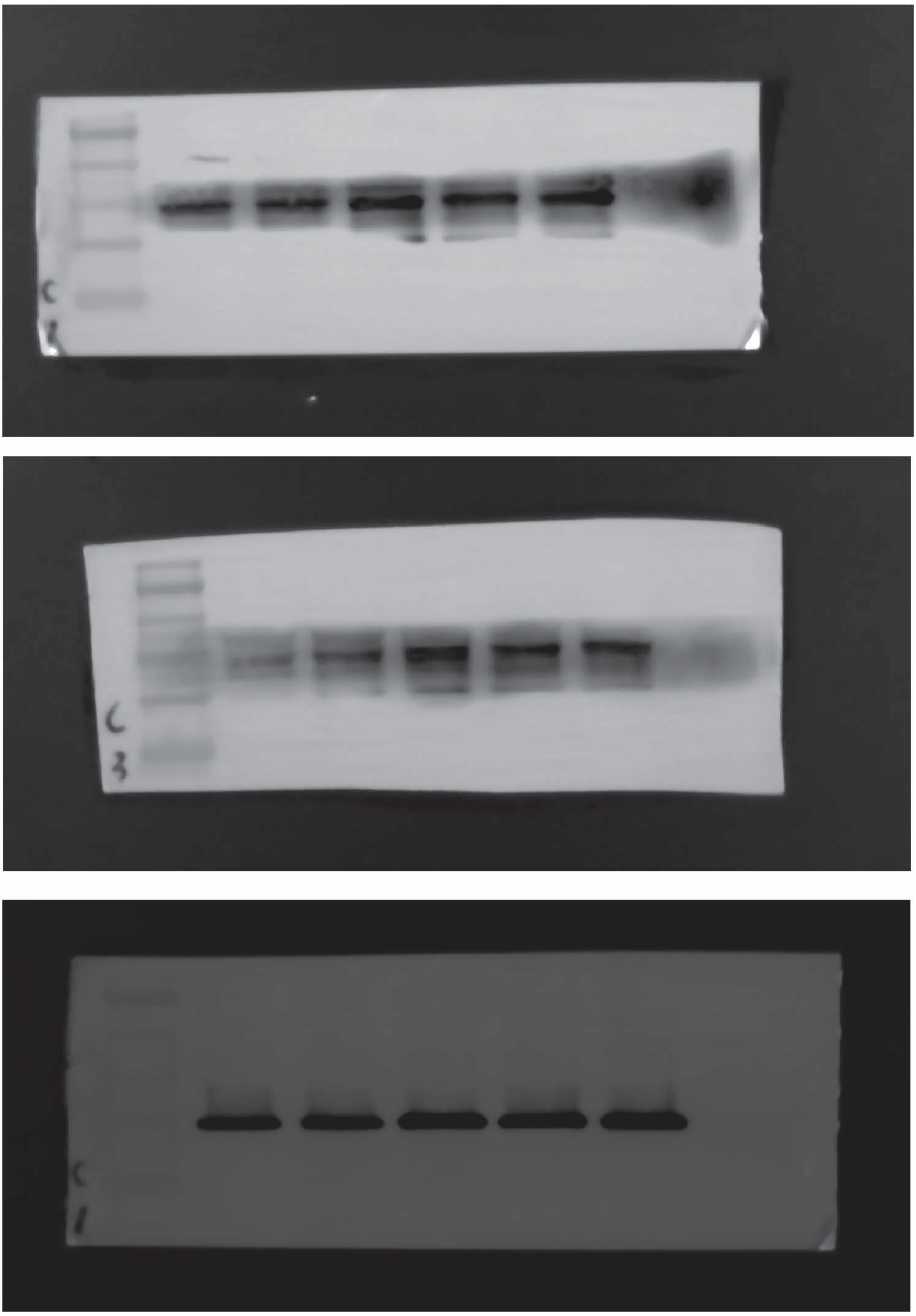

Supplement: Supplementary file 1 — Data S1. [file CNS-31-e70246-s001.pdf]
